# Influence of Plant Physical and Anatomical Characteristics on the Ovipositional Preference of *Orius sauteri* (Hemiptera: Anthocoridae)

**DOI:** 10.3390/insects12040326

**Published:** 2021-04-06

**Authors:** Liu Zhang, Zifang Qin, Pingping Liu, Yue Yin, Gary W. Felton, Wangpeng Shi

**Affiliations:** 1 Department of Entomology and MOA Key Laboratory of Pest Monitoring and Green Management, College of Plant Protection, China Agricultural University, Beijing 100193, China; zhangliucau@163.com (L.Z.); zifangqin351@cau.edu.cn (Z.Q.); liudoubleping@163.com (P.L.); 15851075264@163.com (Y.Y.); 2 Department of Entomology, Pennsylvania State University, University Park, PA 16802, USA; gwf10@psu.edu

**Keywords:** Anthocoridae, noncrop plants, oviposition preference, stomata, trichomes

## Abstract

**Simple Summary:**

The minute pirate bug *Orius sauteri* is the predator of many soft-body pests and has great application prospect in pest suppression in Asia. Females need to insert their ovipositor into plant tissues to lay eggs. Thus, understanding its egg-laying preferences and creating it a beneficial habitat is important for its conversation in the field. We evaluated the ovipositional preference of the females for four noncrop plant species and how the plant characteristics influenced the ovipositional behavior of *O. sauteri*. Our results suggested that *O. sauteri* females were able to select oviposition host and specific sites through assessing the structural qualities of plants. Females were found to prefer plants with high stomatal density, a large stomatal area, and fewer trichomes as oviposition hosts, and the depth of egg placement was determined by leaf thickness. Coriander and marigold are potential oviposition plants for *O. sauteri* for high fecundity and egg hatchability. The results are helpful for selecting beneficial cover crops to natural enemies in the field and lead to a positive outcome for biological control.

**Abstract:**

Natural enemies play an important role in managing insect pests. *Orius sauteri* (Poppius) (Hemiptera: Anthocoridae), a predator of many soft-body insects, is an important biological control agent in Asia. Understanding this predator’s egg-laying preferences and a habitat needs is important for its success in pest control. We investigated the plant acceptability and ovipositional preference of *O. sauteri* for coriander (*Coriadrum sativum* L., Apiales: Apiaceae), marigold (*Tagetes erecta* L., Asterales: Asteraceae), sweet alyssum (*Lobularia maritima* L., Brassicales: Brassicaceae), and alfalfa (*Medicago sativa* L., Fabales: Fabaceae), and focused on the effects of plant physical and anatomical characteristics on the ovipositional preference of *O. sauteri*. The results showed that *O. sauteri* can lay eggs on uninfested plants in the vegetative stage and their eggs hatched normally. *Orius sauteri* females prefer plants with high stomatal density, a large stomatal area, and fewer trichomes as oviposition hosts, and the depth of egg placement was determined by leaf thickness. Our studies suggested that *O. sauteri* females can select oviposition hosts and specific oviposition sites by assessing the structural qualities of plant surface. Coriander and marigold are potentially suitable host plants for *O.sauteri.* The results aid the selection of cover crops to enhance natural enemies in the fields.

## 1. Introduction

Flower bugs (Anthocoridae) are widely used as biological control agents for the suppression of soft-bodied pests such as aphids, psyllids, thrips, and eggs or larvae of Lepidoptera, Coleoptera, and Diptera [1,2,3,4]. The flower bug *Orius sauteri* (Poppius) (Hemiptera: Anthocoridae) is an important biological control agent for agricultural and forestry insect pests in Asia [5], and it has great potential for mass production and field applications.

Noncrop plants, referred to as banker plants, companion plants, or cover plants, have the potential to attract and improve the fitness, abundance, and efficacy of biological control agents for pest regulation [6,7,8,9,10]. These non-crop plants can benefit natural enemies by providing supplementary food resources, suitable microhabitats, and overwintering habitats [11]. The nectar and pollen from plants can be an extra supplemental food source that benefits the survival and reproduction of many natural enemies. Blaauw and Isaacs (2012) found that plots with bigger flowering resources resulted in significantly more natural enemies and greater pest suppression than in smaller flower plots or mown grass areas [12]. The enemy density, group richness, and diversity of natural enemy groups increased with plot size, while the aphid (*Aphis glycines* Matsumura) colonies were smaller as plot size increased. Cornflower *Centaurea cyanus* L. (Asterales: Asteraceae) was attractive to *Microplitis mediator* (Haliday) (Hymenoptera: Braconidae) [13,14], and a significant increase of larval parasitism of *Mamestra brassicae* L. (Lepidoptera: Noctuidae) by *M. mediator* in cabbage (*Brassica oleracea* L., Brassicales: Brassicaceae) fields occurred when *C. cyanus* was added as a companion plant [15]. Flowering coriander increased the abundance of predaceous arthropods, especially flower bugs and coccinellids, when used as an intercrop in crop fields [16,17]. Marigold is a potential attractive crop to natural enemies and can help with biological control of pests [18]. According to Sampaio et al. (2008), that plant hostages species of *Orius* (Hemiptera: Anthocoridae) [19], and flowering marigold was attractive to *O. sauteri* in laboratory and field trials [20]. Sweet alyssum is very attractive to natural enemies and has the potential to be a good ovipositional site for *Orius* species. Sweet alyssum has a long flowering period [21], and its use enhanced densities of predatory Hemiptera in an adjacent tomato field when used as a border planting [22]. Its use as a banker plant for *Orius laevigatus* (Fieber) (Hemiptera: Anthocoridae) has also been suggested [23]. Pumariño and Alomar (2012) suggested alyssum for the conservation of *Orius majusculus* (Reuter) (Hemiptera: Anthocoridae) [24]. Alfalfa has been investigated as an intercrop in cotton fields to aggregate predatory arthropods, such as spiders and ladybirds [25]. Alfalfa adjacent to apple orchards serves as a reservoir for *Orius insidiosus* (Say) (Hemiptera: Anthocoridae) throughout the season [26], and its pollen is attractive to *O. sauteri* [27].

In addition to being potential food resources, plant volatiles may attract beneficial insects from a distance [28,29]. For example, the Asian lady beetle *Harmonia axyridis* (Pallas) (Coleoptera: Coccinellidae) is attracted to nonanal, a volatile from Chinese pagoda tree, *Sophora japonica* L. (Fabales: Fabaceae) [30]. Females of the braconid *Cotesia vestalis* (Haliday) (Hymenoptera: Braconidae) can orientate to nearby flowers of *Brassica rapa* L. (Brassicales: Brassicaceae) via olfactory cues [31]. Plants attacked by herbivores often emit large amounts of different herbivore-induced plant volatiles (HIPVs). Methyl salicylate (MeSA) is a ubiquitous HIPV that is often induced by feeding of herbivores such as mites, aphids, and beetles [32,33,34,35], and HIPVs have been proven to be attractive to various beneficial arthropods, including insect predators, in the agricultural system [36].

For natural enemies, the established capacity to discriminate among odors and choose suitable oviposition sites is important for their successful reproduction and is the key to the field manipulation of natural enemies. Because plants are in flower for only limited periods of time, it is important to increase the use of the plants’ vegetative stage by natural enemies. Rebek et al. (2005) showed that the vegetative characteristics of plants rather than flowers influenced the abundance of natural enemies by the removal of flowers from companion plants [6]. For example, it is known that females of *Cycloneda sanguinea* (L.) (Coleoptera: Coccinellidae) orient towards volatiles from uninfested coriander plants in the vegetative stage [37], suggesting that certain plant species may improve a crop field’s attraction for predators when interplanted with the crop early, while the companion plant is in its vegetative stage.

Previous studies have shown that various plant morphological features can affect the ovipositional behavior and fitness of insects. Trichomes, for example, can block insect movement and serve as a physical and sometimes chemical barrier to insect feeding [38]. The plant epidermis can also have negative effects on the oviposition of flower bugs [39,40].

In this study, we compared coriander, marigold, sweet alyssum, and alfalfa regarding their acceptability to *O. sauteri* for oviposition and the predator’s preference among these plants. We evaluated the potential of these plants as host for *O. sauteri* and investigated potential factors affecting the plant species as oviposition substrates.

## 2. Materials and Methods

### 2.1. Predator Rearing

Colonies of *O. sauteri* were initiated with insects collected from corn fields at the China Agricultural University, Beijing, China in 2015. New males of *O. sauteri* from the field were added to the laboratory colony every year. *Orius sauteri* bugs were reared in 4 L glass jars, and the top of the jars was covered with nylon mesh to allow for ventilation. Inside the jars, buckwheat hulls were added to provide shelter, and eggs of the grain moth *Sitotroga cerealella* (Olivier) (Lepidoptera: Gelechiidae) stuck on Post-it notes were supplied as food. The colony was maintained at 27 ± 1 °C, 55 ± 5% relative humidity (R.H.), and a 16 h:8 h light (L)/ dark (D) photoperiod. We provided kidney bean pods as an oviposition substrate for adults. Bean pods were removed every 2 days and transferred to new jars for rearing of the nymphs. Once the adults emerged, females and males were reared together. To ensure the females mated, we used *Orius sauteri* females that 6 ± 1 days old that had been held in a mixed-sex colony for experiments.

### 2.2. Plant Material

All plants used in experiments were grown in a chamber under controlled temperature and humidity (26 ± 1 °C, 70 ± 5% R.H.) in plastic pots (diameter = 14 cm, height = 7 cm) filled with potting soil and watered once per week. There were 5 plants in each pot. One month later, the plants were used in assays when they reached the following stages: Corianders, with 4 true leaves, and marigolds, sweet alyssum, and alfalfa, with 6 true leaves.

### 2.3. Behavioral Preferences of Minute Pirate Bug for Different Plant Species

A behavioral experiment was run to test the response of adult females to the 4 test plant species. Coriander, marigold, sweet alyssum, and alfalfa were transplanted as individual plants into soil-filled plastic cups (diameter = 5 cm, height = 7 cm), and then plants were allowed to recover for 48 h in the incubator. The cup was covered in metal foil, and then the 4 different plants were positioned in 4 corners of the test arena (30 × 30 × 30 cm). Then, 24 female *O. sauteri* adults were placed in the center of the cage and allowed to move freely. Following this, the number of females located on each of the 4 plant species was checked after 30 min, 1 h, 2 h, and 4 h. Seven replicates were performed.

### 2.4. Plant Acceptability and Oviposition Preferences

#### 2.4.1. Plant Acceptability

We used a no-choice design. Considering the differences in size among the four plant species being tested, we considered 1 experimental unit to be 1 marigold, 1 sweet alyssum, 2 coriander plants, and 3 alfalfa plants, which were used in those numbers in each assay. All test plants were removed from their soil carefully in order to avoid any unnecessary damage. Plant roots were then provided with absorbent cotton and distilled water for moisture. Then, the plant materials were placed in plastic cups, 1 species per container (1 L) (Figure S1A,B). The experimental setup followed Waite et al. (2014) [41], with some modifications. Plants were allowed to recover for 48 h in an incubator with constant temperature and humidity (26 ± 1 °C, 70 ± 5% R.H., 16L/8D). Five mated female bugs were then placed in each container and were allowed to oviposit for 48 h at 26 ± 1 °C, 30 ± 5% R.H., and 16L/8D. A piece of Post-it note with grain moth egg was placed in the container and served as food. The rim of the container was covered with nylon mesh for airflow. After 48 h, plants were then observed under a stereoscope, and the number of eggs laid in the plant(s) in each container were recorded. Twelve replicates were carried out.

#### 2.4.2. Oviposition Preferences

We measured preference for oviposition in a multiple-choice assay. The plant materials were prepared as described above. However, all 4 plant species were placed in 1 container (1 L) (Figure S1A,C), and the positions of the 4 plant species were random. Five mated females were introduced to each container and allowed to oviposit for 48 h at 26 ± 1 °C, 30 ± 5% R.H., and 16 h:8 h L/D. A piece of Post-it note with grain moth egg was placed in the bottom center of the container, which ensured 4 plant species at the same distance from the food. After 48 h, the number of eggs on each species of plant was counted under a stereoscope. There were 11 replicates of this experiment.

### 2.5. Egg Hatch on Different Plant Species

Plant tissues with eggs were collected from the no-choice oviposition assay and placed in different Petri dishes (diameter = 9 cm) for each plant species. A layer of 2.5% agar was placed in Petri dishes to keep plant tissues hydrated during the observation period. Each Petri dish contained 17–21 eggs, and a total of 6 replicates per plant species were used. The Petri dishes were kept in an incubator (26 ± 1 °C, 70 ± 5% R.H.) to observe egg hatch. The number of newly emerged nymphs in each dish were counted and removed every day until all the eggs hatched or no nymphs appeared for 2 additional days.

### 2.6. Plant Morphological Characteristics

Stems and leaves of different plant species from Section 2.4 that contained *O. sauteri* eggs and without eggs were cut off with a razor blade and fixed overnight in 2.5% glutaraldehyde prepared in 0.1 M potassium phosphate buffer (PPB). Then, samples were washed with distilled water and then further dehydrated in different ethanol series (30, 50, 70, and 90%) for 15 min individually, dried with critical evaporator (Leica EM CPD030, Leica Microsystems, Germany), and coated with gold for observation. The lower surface of leaves and stems were then observed with a scanning electron microscope (HITACHI SU8010, Japan). The number of trichomes and stomata on tissues under 1 field of view (1 image) was counted at a voltage of 10 kV and 8.00 mm ×250 LM. We measured the stomatal area of the different plants by ImageJ 1.51j8 (Wayne Rasband, National Institutes of Health, Bethesda, MD, USA). For the leaves, we measured 5–6 stomata around egg-laying sites in each sample. We also measured stomata on stems, which had fewer stomata than leaves.

Paraffin-based sectioning was used to investigate internal anatomical characteristics among the plant species. Plant tissues containing eggs were removed with a razor blade and preserved in formalin/acetic acid/alcohol (FAA, 50% alcohol) fixative. Cross-sections of plant tissues with *O. sauteri* eggs were sectioned to a thickness of 5 μm on a microtome and stained with safranin and counterstained with fast green solution [42]. Slides were examined using Zoom-stereo microscope (Chongqing Optec, China) and photographed at X10 using an industrial digital camera (OPTEC CCD TP510, Chongqing Optec, Chongqing, China). We measured the leaf’s blade cross-section thickness (adjacent to egg-hatch sites), the depth of egg deposition, and the minimum distance from the epidermis to vascular tissues of stems for the different plant species. Each sample was measured 3 times, except for leaf thickness, which consisted of 6 measurements.

### 2.7. Statistical Analysis

All data were analyzed with IBM SPSS 25 (IBM Corp., Armonk, NY, USA). The preference of *O. sauteri* females among the 4 plant species over time was analyzed using a two-way analysis of variance (ANOVA) with α = 0.05. The number of females staying on each of the different plant species for 30 min were compared using ANOVA, followed by a LSD (Least Significant Difference) test with α = 0.05. For the plant acceptability and oviposition preferences tests, the number of eggs per plant species in the no-choice assay and the multiple-choice assay were compared using one-way ANOVA (LSD, α = 0.05) and the Kruskal–Wallis nonparametric test, respectively. The hatch rate of eggs among different plant species were compared by the Kruskal–Wallis nonparametric test.

For the morphological features of plants, the trichome densities on the same plant tissue on preferred and non-preferred sites were compared separately for each plant species using *t*-tests. The stomatal densities and areas of leaves and stems among 4 plant species were analyzed by one-way ANOVA (LSD, α = 0.05). For the internal features, the thickness of leaves and minimum distance from the epidermis to the vascular tissue of stems were analyzed by ANOVA (LSD, α = 0.05).

## 3. Results

### 3.1. Behavioral Preferences

**Figure 1:**
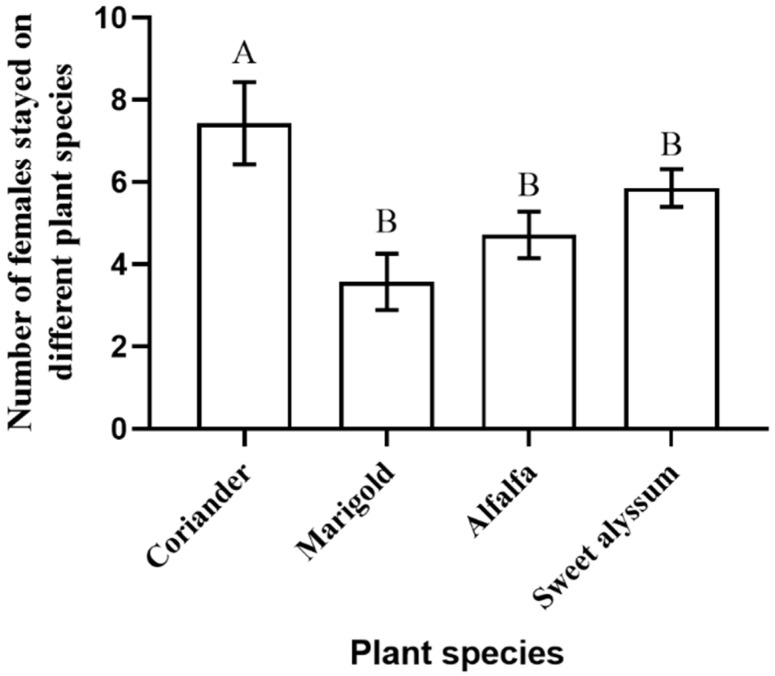
The preference of *Orius sauteri* females among four plant species after 30 min (*n* = 11; α = 0.05; ANOVA). Values are mean ± SE (Standard Error). Means indicated by the same letter are not significantly different according to Least Significant Difference (LSD) test.

Plant species have a significant effect on *O. sauteri* choice for resting location (F_3_ = 16.526, *p* < 0.001), while experiment duration did not affect (F_3_ = 0.083, *p* = 0.994) (Table 1). Females had a strong preference for coriander compared to the other three plant species (30 min: F_3,27_ = 5.444, *p* = 0.005; Figure 1).

### 3.2. Plant Acceptability and Ovipositional Preferences for Different Kinds of Plants

**Figure 2:**
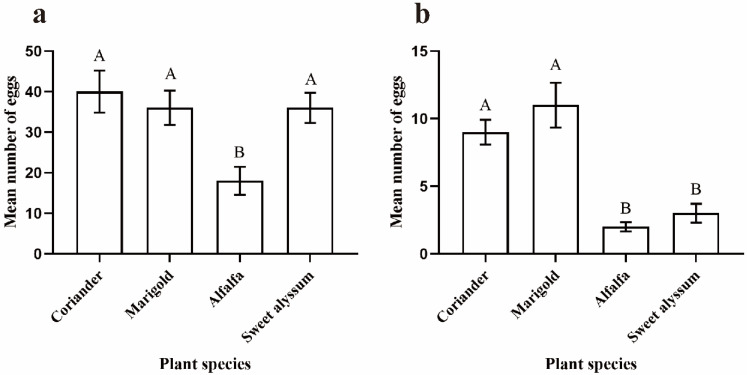
Comparison of the number of *Orius sauteri* eggs laid among different plant species. (**a**) Single-choice assay (*n* = 12; α = 0.05; ANOVA); (**b**) multiple-choice assay (*n* = 11; α = 0.05; Kruskal–Wallis nonparametric comparison). Values are mean ± SE (mean: Egg amount of five females in 48 h). Means indicated by the same letter are not significantly different according to LSD test.

All *O. sauteri* females survived during the experimental period. The numbers of eggs laid on coriander, marigold, and sweet alyssum were greater than that on alfalfa in the no-choice assay (F_3,47_ = 5.650, *p* = 0.002; Figure 2a). The females laid 40 ± 5.2 eggs (egg amount of five females in 48 h) in the coriander plant. There were an average of 36 ± 4.2 eggs and 36 ± 3.7 eggs found in marigold and sweet alyssum plants, respectively. Alfalfas was less acceptable than the other three plant species with 18 ± 3.4 eggs.

*Orius sauteri* showed a significant ovipositional preference in the multiple-choice assay (H _(3,*n* = 44)_ = 29.558, *p* < 0.001; Figure 2b), with significantly more eggs laid on coriander (9 ± 0.9 eggs) than sweet alyssum (3 ± 0.7 eggs) (H = 17.682, *p* = 0.007) or alfalfa (2 ± 0.3 eggs) (H = 21.045, *p* = 0.001). Egg numbers on marigold (11 ± 1.7 eggs) were also higher than on sweet alyssum (H = 20.409, *p* = 0.001) or alfalfa (H = 23.773, *p* < 0.001).

### 3.3. Egg Hatch on Different Plant Species

**Figure 3:**
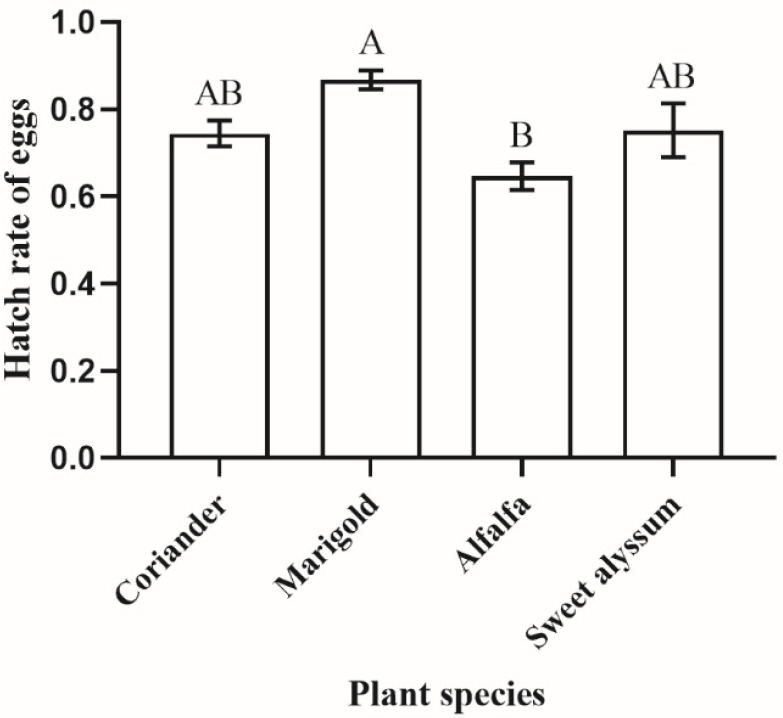
The hatchability of *Orius sauteri* eggs on different plant species (*n* = 6; α = 0.05; Kruskal–Wallis nonparametric comparison). Values are mean ± SE. Means indicated by the same letter are not significantly different.

The egg hatch rate on different plant species showed significant differences (H = 9.309, *p* = 0.025; Figure 3). The highest hatch rate was found on marigold (87 ± 2%), and the lowest was on alfalfa (65 ± 3%). The hatch rate of the eggs from marigold was significantly higher than that of alfalfa. However, the hatch rate of the eggs from coriander and sweet alyssum was not significantly different from that of marigold and alfalfa.

### 3.4. Effects of Plant Morphological Characteristics

**Figure 4:**
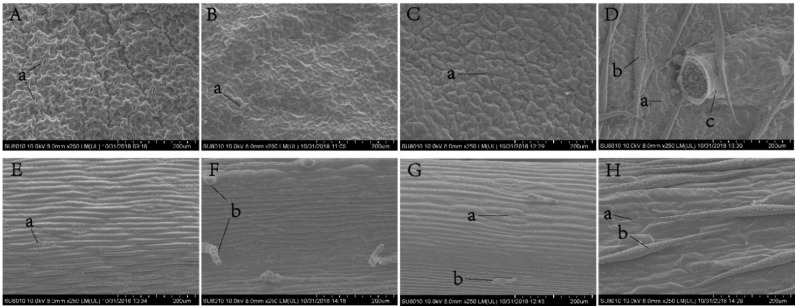
The surface character of four plant species: (**A**) coriander leaf; (**B**) marigold leaf; (**C**) alfalfa leaf; (**D**) sweet alyssum leaf; (**E**) coriander stem; (**F**) marigold stem; (**G**) alfalfa stem; (**H**) sweet alyssum stem; a: Stomata; b: Trichomes; c. *Orius sauteri* eggs in sweet alyssum leaves; magnification: 250×.

**Figure 5:**
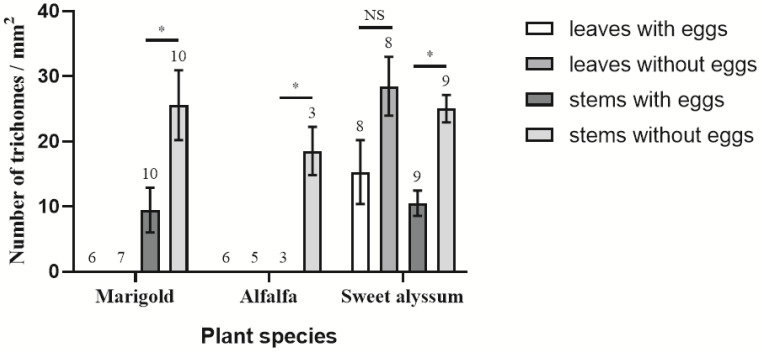
A comparison of the trichome densities on oviposition sites and sites without eggs on the stems and leaves of different plant species. The trichome densities of the same plant tissue with and without *Orius sauteri* eggs were compared separately for each plant species. * Indicates significant differences (α = 0.05; *t*-test) * *p* < 0.05. Sample sizes for each plant species are indicated above the bars. Values are mean ± SE.

Females deposited eggs into the plant tissues (Figure 4C,D). The egg caps projected above the surface, affording an exit for the hatchlings. Trichomes on different plant species varied in their distribution and density (Figure 4). Trichomes were abundant on the lower surface of sweet alyssum leaves, but there were no trichomes on the surface of coriander plants or the lower surface of marigold leaves. A small number of trichomes were present on the main leaf vein of alfalfa leaves and few eggs were found near veins with trichomes in our experiments. Consequently, we compared the trichome densities of the plant tissues of a given species with and without *O. sauteri* eggs. This comparison was made for each plant species except for coriander. Significantly more trichomes were found at sites without eggs than sites with eggs on stems of marigold (t_18_ = 2.126, *p* = 0.021), alfalfa (t_2_ = 16, *p* = 0.038), and sweet alyssum (t_15_ = 0.587, *p* < 0.001; Figure 5). The density of trichomes surrounding oviposition sites was lower than at sites without eggs on the lower surface of sweet alyssum leaves (t_12_ = 0.0.663, *p* = 0.068).

**Figure 6:**
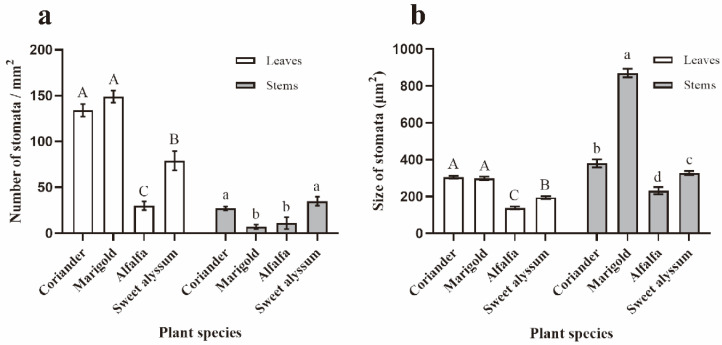
A comparison of the stomatal densities (**a**) and size (**b**) of leaves and stems among four plant species (α = 0.05; ANOVA). (**a**) Stomatal densities, sample sizes for each plant species: leaves *n* = 5–11; stems *n* = 3 for alfalfa, *n* = 7–10 for other three plant species. (**b**) Stomatal densities. Sample sizes for each plant species: leaves *n* = 23–25, stems *n* = 8–20. Values are mean ± SE. Means indicated by the same letter are not significantly different according to LSD test.

Stomatal densities were significantly different among the different plant species on both leaves and stems (leaves: F_3,30_ = 27.801, *p* = 0.007; stems: F_3,27_ = 14.440, *p* < 0.001). The densities of stomata on the lower surfaces of coriander or marigold leaves were significantly higher than on leaves of alfalfa or sweet alyssum. The stomatal densities of coriander and sweet alyssum stems were significantly higher than those stems of marigold and alfalfa (Figure 6a).

There were significant differences in stomatal areas of leaves and stems for the four plant species (leaves: F_3,118_ = 89.592, *p* < 0.001; stems: F_3,53_ = 215.916, *p* < 0.001). The stomatal areas of coriander and marigold were significantly larger than those of alfalfa and sweet alyssum (Figure 6b).

**Figure 7:**
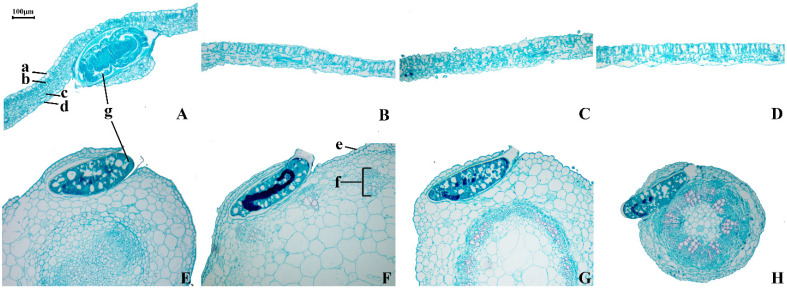
Cross-section of leaves and stems of four different plant species. (**A**) Coriander leaf; (**B**) marigold leaf; (**C**) sweet alyssum leaf; (**D**) alfalfa leaf; (**E**) coriander stem; (**F**) marigold stem; (**G**) sweet alyssum stem; (**H**) alfalfa stem; (a) upper epidermis; (b) palisade tissue; (c) spongy tissue; (d) lower epidermis; (e) epidermis; (f) vascular tissue; (g) eggs of *Orius sauteri*. Scale bars: A-H, 100 μm. Magnification: 100×.

**Figure 8:**
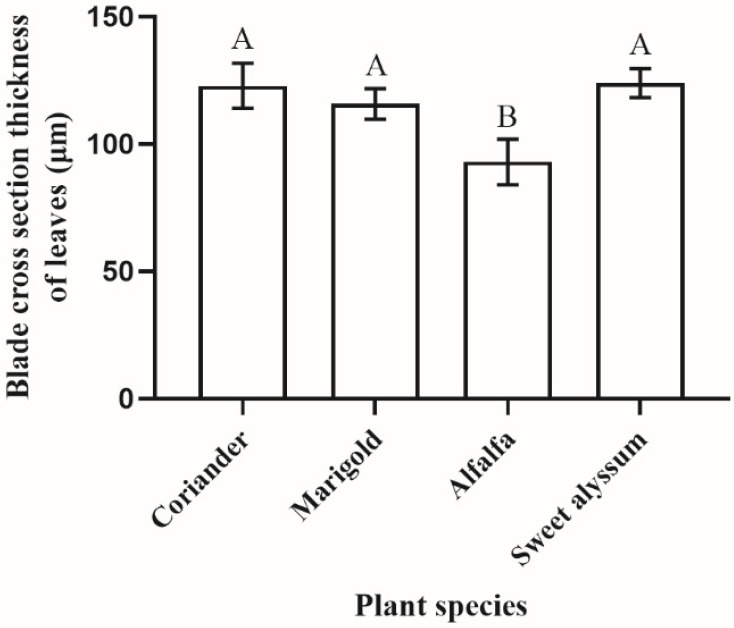
A comparison of the thickness of leaf-blade cross-sections among different plant species (*n* = 5–7), indicating significant differences (α = 0.05; ANOVA). Values are mean ± SE. Means indicated by the same letter are not significantly different according to LSD test.

**Figure 9:**
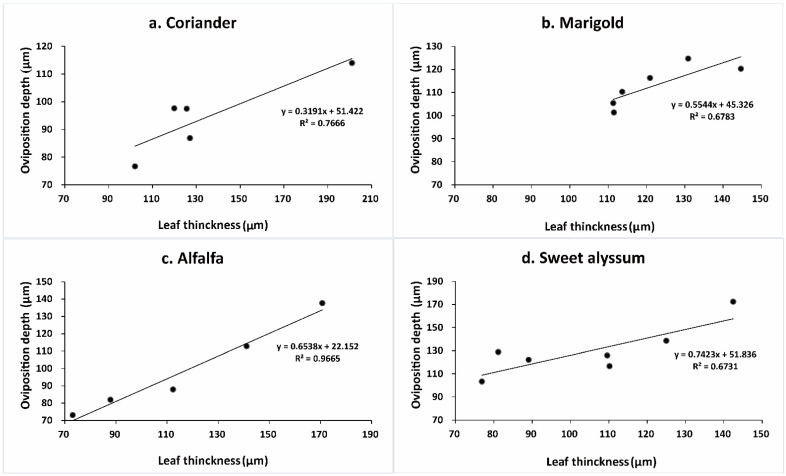
The relationship of depth of *Orius sauteri* egg placement and leaf thickness.

Internal anatomy of plant tissues also varied among plants is ways that affected *Orius* bugs. Under the upper epidermis of leaves is a dense palisade tissue composed of vertically arranged cells, while under the lower epidermis is spongy tissue composed of 4–5 layers of sparse random arrangement cells (Figure 7A–D). The thickness of leaves of the test plant species differed significantly (F_3,23_ = 3.488, *p* = 0.035; Figure 8). The leaves of coriander, marigold, and sweet alyssum were much thicker than those of alfalfa. The depth of egg placement was correlated with the leaf thickness, except for coriander (marigold: *R^2^* = 0.829, *p* = 0.041; alfalfa: *R^2^* = 0.983, *p* = 0.003; sweet alyssum: *R^2^* = 0.820, *p* = 0.024; coriander: *R^2^* = 0.876, *p* = 0.052; Figure 9).

**Figure 10:**
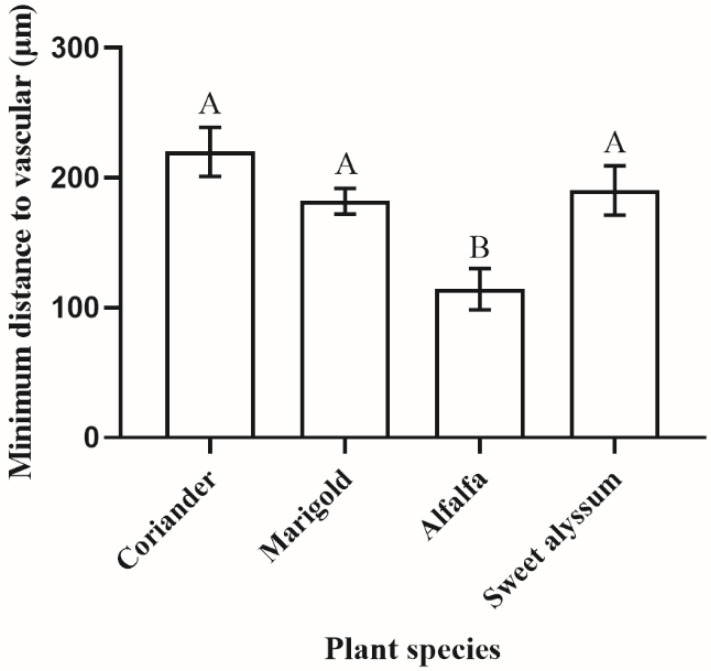
Minimum distance from the epidermis to vascular tissue of stems in different plant species near oviposition sites of *Orius sauteri* (*n* = 3 for alfalfa, *n* = 6 for other plant species) (α = 0.05; ANOVA). Values are mean ± SE. Means indicated by the same letter are not significantly different according to LSD test.

The cross-sections of stems for the four plant species had similar anatomical details with epidermis, collenchyma, phloem, and xylem (Figure 7E–H). We found that females avoided penetrating vascular bundles when laying their eggs. In alfalfa, the minimum distance from the epidermis to the vascular tissue was significantly shorter than for the other three plant species. (F _3,19_ = 6.653, *p* = 0.004; Figure 10).

## 4. Discussion

Our experiments showed that the physical and anatomical characteristics of plants, especially stomatal density, stomatal area, trichome density, and tissue thickness, affected the ovipositional preferences of *O. sauteri*. Females displayed clear preference for laying eggs on coriander and marigold compared to sweet alyssum or alfalfa. *Orius sauteri* laid the fewest eggs on alfalfa regardless of the presence or absence of alternative plant species in our study. Zhou (1991) also found that *O. sauteri* has a preference for certain plant species or tissues for oviposition [43]. Unlike previous studies [41,43,44], we offered whole plants to females instead of just part of the plant. Thus, *O. sauteri* was able to identify better egg-laying sites, but the mechanisms behind such oviposition decisions and the benefit for biological control remain to be further investigated.

Because *O. sauteri* females need to insert their ovipositors into plant tissues to lay eggs, the physical and anatomical characteristics of plants may affect ovipositional choices. *Orius* females can distinguish degrees of epidermal thickness and trichome density on an extremely fine scale and lay eggs at specific sites in plants [39]. Here, we found that *O. sauteri* females preferred the plants with a higher density of stomata (and a larger stomatal area) and with fewer trichomes for oviposition. Sweet alyssum has a high density of long trichomes, which resulted in ovipositional avoidance of this plant in our laboratory choice tests. Typically, *O. sauteri* chose oviposition sites on plants with fewer trichomes. Armer et al. (1999) suggested that trichomes hindered the egg-laying process [45]. The effect of epidermal thickness in our experiment was small because entire plants were used, with the result that oviposition sites of varying thickness were available on all plant species tests. Stomatal number and size can also affect the ovipositional preference of *O. sauteri*. The flower bug *Elatophilus hebraicus* Pericart (Hemiptera: Anthocoridae) usually oviposits through stomata, which may make ovipositional insertion easier [46]. We observed in our study that *O. sauteri* preferred to lay eggs on coriander and marigold, species with high stomatal densities and area. In addition, vascular bundles may be a barrier to oviposition, mainly because of the presence of xylem, which is lignified and thus difficult to penetrate. In alfalfa, the minimum distance between the surface to vascular bundles of the stem was significantly shorter than for the other three plant species, and the distance maybe not sufficient to house an egg. Similarly, females preferred thicker leaves to oviposit for sufficient room for eggs. Although a limited number of samples were measured, it still makes sense that the depth of egg placement was correlated with the leaf thickness of marigold, alfalfa, and sweet alyssum.

Additionally, both *O. insidiosus* and *Orius minutus* L. prefer to lay eggs in the tender parts of the host plant because these tissues are likely easier to puncture [47,48]. Likewise, *O. sauteri* also tended to oviposit on tender plant tissues. *Orius sauteri* made significantly more ovipositor punctures and laid more eggs on sprouts compared to leaves of kidney beans, and sprouts proved to be a superior substrate for oviposition [44,49]. Our studies showed that *O. sauteri* tends to lay its eggs in thicker leaves and stems with a longer distance between the epidermis and vascular bundles. The stems of alfalfa were thinner and tougher and received fewer eggs than the other plants we tested.

Plant acceptability for oviposition may also be affected by the plant’s suitability for egg hatching and subsequent development of *Orius* nymphs [40]. According to the optimal oviposition theory, insects should oviposit on plants that can benefit their progeny most [50,51]. For example, Tawfik and Ata (1973) found *Orius albidipennis* (Reuter) oviposited more in the calyx or near flowers of its asteraceous host plants [52]. *Orius insidiosus* also laid more eggs on the calyx or flower petiole when the plant had a single flower [53] because the bug’s preferred prey, thrips such as *Frankliniella occidentalis*, have nymphs that live in flowers [54,55]. Oviposition preferences of females should be in line with host plant suitability for offspring development because females need to lay their eggs on high-quality hosts to maximize their fitness [56]. For example, Zhang et al. (2021) found that red bean was a suitable banker plant of *O. sauteri* in tea plantation because of its good performance in supporting the development of nymph and reproductive capacity of adults [57]. However, oviposition sites also must function as suitable shelters for eggs, the oviposition substrate must support both a high egg hatch rate and provide resources for development of the resulting nymphs or avoidance of egg parasitoids. In our study, we found that plant species had a significant influence on egg hatch, specifically the hatch rate of *O. sauteri* eggs was much lower on alfalfa than the other three plant species tested.

The availability of suitable oviposition sites for insect predators can be important in biological control programs, and the number of such sites could be enhanced through the increased use of plant diversity in or near crops [58]. Zhao et al. (2017) found that the addition of *Calendula officinalis* L. (Asteraceae) plants in greenhouse crops enhanced predator populations [5]. However, our knowledge of the selection of suitable host plants for promotion of higher *O. sauteri* densities is still limited. The results of our work suggest that coriander and marigold are potential oviposition plants for *O. sauteri*, and their use might enhance flower bugs in the field.

The effects of plant physical and anatomical characteristics on the ovipositional preference of *O. sauteri* were important at close range. However, there are many other factors, such as plant semiochemicals and abiotic factors, that affect the oviposition/development of *O. sauteri* on the host plants in the field condition. According to Groenteman et al. (2006), oviposition behavior was influenced by the nitrogen level of host plants, and females of *O. albidipennis* showed significant defensive behavior to obtain a better ovipositional site on nitrogen-rich plants than nitrogen-poor plants [59]. Besides the plant volatiles [28,29,30,31], volatiles released by conspecifics or other insects also have effects on oviposition or development of minute pirate bugs. Studies found that they take advantage of kairomone emanating from their prey [60,61] to benefit their offspring. Moreover, we know that *O. insidiosus* and *O. sauteri* exhibit a pronounced sexual dimorphism of the metathoracic scent gland secretion [62,63]. Females of *O. insidiosus* produce a volatile sex pheromone and a non-volatile trail pheromone. *Orius insidiosus* males most likely respond to the trail pheromone as the ultimate means to locate potential mates, whereas the benefit of females responding to the trail pheromone may be that this signal acts as a cue indicating the likelihood of finding nearby prey [62]. We excluded the influence of males in our study by only placing mated females into the container. However, there are still many semiochemical investigations of *O.sauteri* that are needed to help us understand female oviposition behavior and realize a successful population establish in the field.

In summary, our results suggest that *O. sauteri* females can select oviposition host plants and specific sites on plants through assessment of their structural characteristics. Females of *O. sauteri* prefer plants with high stomatal density, large stomatal areas, and low trichome densities as oviposition hosts. The physical and anatomical characteristics of plants in our study not only affected the ovipositional preferences of *O. sauteri* but also had an effect on the egg hatching rate, which would be important to the development of an *O. sauteri* population. A limited number of plant species were tested in our study, and evaluations of other plant species or different cultivars of the four plant species are needed further. Moreover, host–plant selection and oviposition of natural enemies is a complex process involving plant morphological characteristics, many plant semiochemicals, or abiotic factors. Further studies on mechanisms behind ovipositional preference and reproduction by *O. sauteri* under field conditions will help to identify specific plant qualities needed to best promote this species’ contribution to biological control.

## Figures and Tables

**Table 1:** Results of two-factor ANOVA for the behavioral preference of *Orius sauteri* females to four plant species over time.

Effects	SS	Df	MS	F	*p*
Plants	245.670	3	81.890	16.526	<0.001
Time	0.384	3	0.128	0.026	0.994
Plants*Time	38.223	9	4.247	0.857	0.566
Error	475.714	96	4.955		

## Data Availability

The data presented in this study are available on request from the corresponding author.

## Supplementary Materials

The following are available online at https://www.mdpi.com/article/10.3390/insects12040326/s1: Figure S1, The container for *Orius sauteri* in the oviposition preferences assay.
