# Supplementary figures and images for: Influence of Plant Physical and Anatomical Characteristics on the Ovipositional Preference of Orius sauteri (Hemiptera: Anthocoridae)
Source: Insects. 2021 Apr 6;12(4):326. doi: 10.3390/insects12040326 (PMC8067476; doi:10.3390/insects12040326)

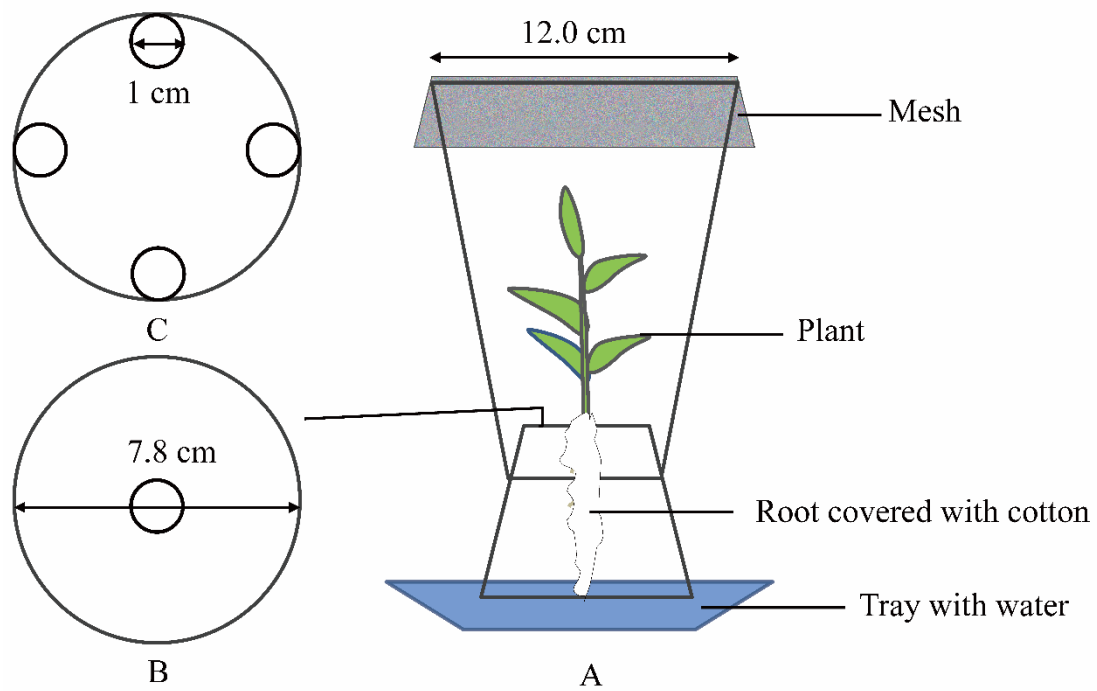

**Figure. S1.** The container for *Orius sauteri* in the oviposition preferences assay.

Supplement: Supplementary file 1 [file insects-12-00326-s001.pdf]
